# The application of qNMR for the determination of rosuvastatin in tablet form

**DOI:** 10.3906/kim-2007-7

**Published:** 2021-02-17

**Authors:** Gökhan DİKMEN, Okan USLU

**Affiliations:** 1 Eskisehir Osmangazi University, Central Research Laboratory Research and Application Center (ARUM), Eskisehir Turkey

**Keywords:** qNMR, proton nuclear magnetic resonance, high performance liquid chromatography, rosuvastatin, validation

## Abstract

In this study, quantative nuclear magnetic resonance (qNMR) method was used to determine the content of rosuvastatin in tablet. Linearity, range, limit of detection (LOD), limit of quantification (LOQ), accuracy, and precision were determined in validation study of rosuvastatin. Furthermore, validation study of rosuvastatin was performed with high performance liquid chromatography (HPLC). Uncertainties of qNMR and HPLC methods were determined using per EURACHEM/CITAC Guide CG 4 (3th edition), quantifying uncertainty in analytical measurement. qNMR and HPLC methods were linear in the ranges of 0.10 - 5.00 mg/mL and 0.001 - 0.0995 mg/mL, respectively and these lineraties indicate very good linearity performance with regression coefficients (R2 value) above > 0.99. Moreover, LOD and LOQ values using qNMR method were observed as 0.25 mg/mL and 0.80 mg/mL, respectively. These values using HPLC method were found as 0.00051 µg/mL and 0.001695 µg/mL, respectively. The strengths and weaknesses of qNMR method and HPLC method were determined with spectral emphasis on the role of identical reference standards in qualitive and quantitative analyses. It was found that qNMR method is simple, efficient, reliable, and accurate method. Moreover, qNMR method is an easy, practical, and useful method for the validation and optimization of rosuvastatin in the tablet.

## 1. Introduction

High performance liquid chromatography (HPLC) and mass spectroscopy have been used as a quantitative analysis method for many years. However, nuclear magnetic resonance (NMR) spectroscopy was developed in recent years to determine contents in the metabolite, drugs, etc., and this method has been described as quantitative nuclear magnetic resonance (qNMR) spectroscopy or qNMR method [1-6]. Although the sensitivity of qNMR method is lower than chromatographic methods, qNMR method has some advantages over chromatographic methods [7]. In order to create calibration graph, qNMR method does not need a pure reference material [8]; qNMR method performs structural characterization instead of destroying the sample, and the analysis of mixtures can be performed without preisolation process using qNMR method. The basis of qNMR is the relationship between integral value of signal and the proton numbers. Moreover, signal to noise ratio of signal can be used to quantification of material. In order to perform more reliable and more accurate quantification of sample, internal standard material can be used. However, the most important point here is that signals of internal standard material and signals of sample are not definitely overlapping with each other [9-13].

**Figure 1:**
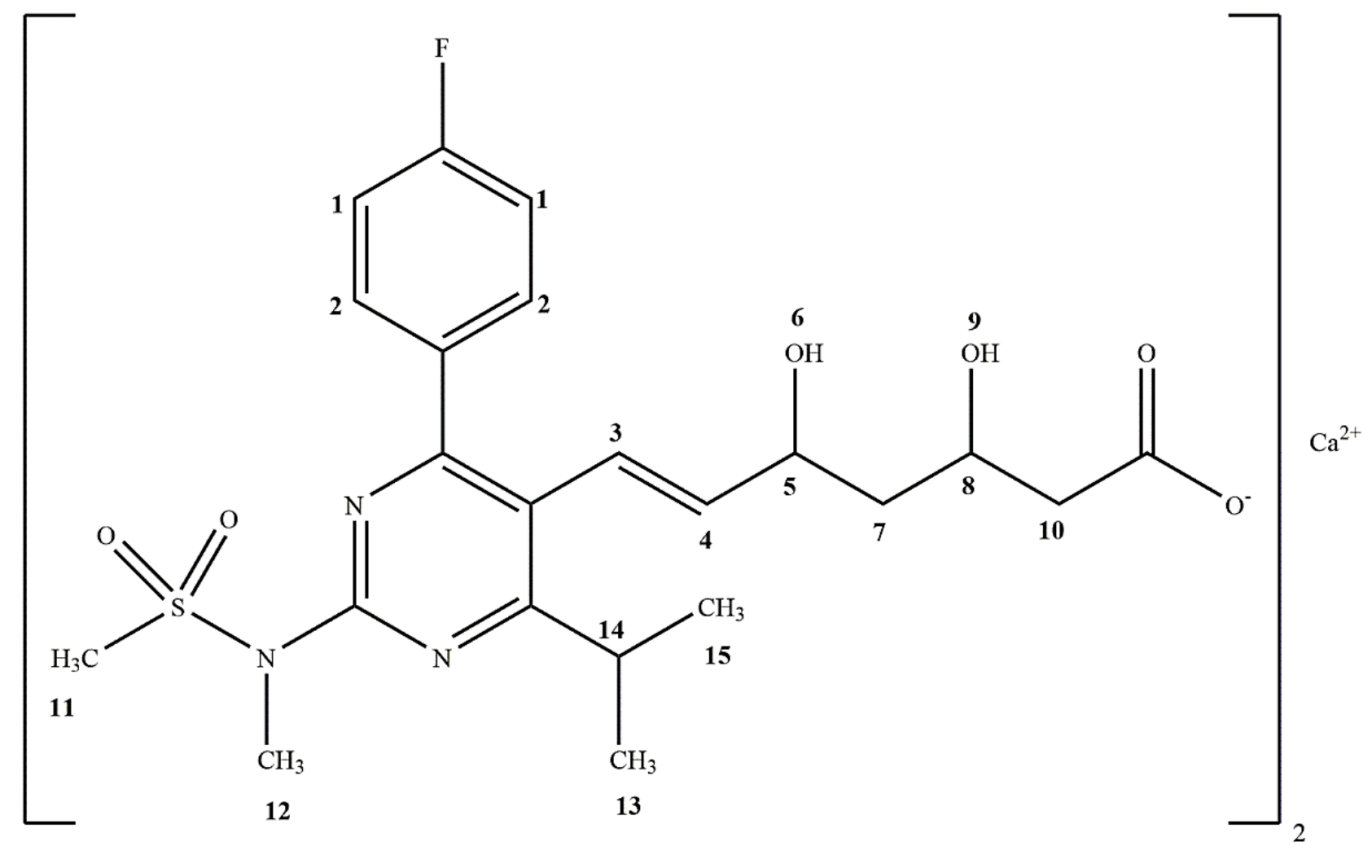
Chemical structure of Rosuvastatin calcium.

Chemical name and empirical formula of rosuvastatin are bis{(E)-7-[4-(4-fluorophenyl)-6-isopropyl-2-[methyl(methylsulfonyl)ami-no]pyrimidin-5-yl](3R,5S)-3,5-dihydroxyhept-6-enoic acid} calcium salt, (C_22_H_27_FN_3_O_6_S)_2_Ca, respectively. Moreover, molecular weight of rosuvastatin calcium is 1001.14 g/mol and chemical structure is given in Figure 1 [14,15]. Rosuvastatin was used to prevent u1d88. Rosuvastatin has been shown to possess a number of advantageous pharmacological properties, including enhanced HMG-CoA reductase binding characteristics and selective uptake into/activity in hepatic cells. In the literature, amount of rosuvastatin in tablets was determined by high-performance liquid chromatography (HPLC) [16-19]. However, any research about determination of amount of rosuvastatin in tablet form using qNMR has not been reported in the literature.

In this study, amount of rosuvastatin in tablet form was determined using qNMR method as an alternative to HPLC. In addition, amount of rosuvastatin in tablet form was examined with HPLC. Excellent quantitative results were shown in the determination of rosuvastatin in tablet and pure rosuvastatin using qNMR. As a result, it is shown that qNMR method is an accurate, precise, and selective method for the determination of the amount of rosuvastatin in tablet.

## 2. Experimental

### 2.1. Chemicals and reagents

Rosuvastatin calcium (98%) and ellagic acid (95%) were purchased from Sigma-Aldrich (St. Louis, MO, USA). Deuterodimethyl sulfoxide (DMSO-d_6_, D 99.3% and 0.03% TMS) was purchased from Cambridge Isotope Laboratories Inc. (Andover, MA, USA). HPLC grade acetonitrile was purchased from VWR International (Bois, France). Ortho-phosphoric acid (85%) was purchased from Merck (Darmstadt, Germany). Ultrapure water was generated from Milli-Q system (Merck, Millipore, Billerica, MA, USA). Colnar tablet containing 5 mg rosuvastatin was bought from Sanovel (İstanbul, Turkey). Standard 5-mm NMR tubes were purchased from Norell Inc. (Landisville, NJ, USA).

### 2.2. Instruments

NMR spectra were collected with JEOL ECZ 500R (JEOL Ltd., Tokyo, Japan) at room temperature and proton resonance frequency was adjusted to 500.13 MHz. Tetra methylsilane (TMS) and DMSO- d_6_were used as internal standard and solvent, respectively. In the qNMR method, pulse angle was used as 90°. Data points, number of scan and spectral width were adjusted as 48 K, 32, and 12.5 kHz, respectively. Relaxation time was adjusted to 25 s. All chemical shifts were reported in parts per million (ppm) relative to dimethyl sulfoxide (DMSO- d6) at 2.51 ppm and TMS at 0.00 ppm. Phase and baseline distortions of spectra were adjusted automatically by JEOL Delta NMR Software (version 5.1.3). Moreover, each NMR experiment was repeated three times including sample preparation.

Thermo Scientific Dionex Ultimate 3000 HPLC (Germering, Germany) system equipped with gradient pump, a variable wavelength programmable PDA detector was used. In the optimized procedure, the separation was performed at flow rate 0.8 ml/min through a Zorbax ODS C18 analytical column (5 µm, 150 mm × 4.6 mm) (GL Science Inc., Japan) and the column compartment temperature was adjusted to 30 ℃. The mobile phases were used as isocratic system. Separation was achieved using a mobile phase acetonitrile: water (40: 60, v/ v) and pH of mobile phase was adjusted at 3.5 with phosphoric acid [20]. Rosuvastatin detection was carried out at 242 nm wavelength. The injection volume was 5 µL and analysis time was 12 min (including reequilibration). All mobile phases were degassed and filtered through 0.2 µm membrane filter (GUS, Standfor, ME, USA) before use.

### 2.3. Preparation of samples

In order to prepare standard solution for qNMR, 0.12, 2.14, 2.93, 4.24, and 4.91 mg (± 0.01 mg) rosuvastatin standard sample and 1.02 mg (±0.01 mg) ellagic acid (internal standard) were weighed using Ohaus AX224 (Ohous Cor., USA), and then these samples were put into the 5 mm NMR tube. 1 mL DMSO- d_6_solvent was added on each sample using pipet tip (Eppendorf, Hamburg, Germany). The solution was mixed until a clear solution was obtained. Each solution was prepared 3 times to obtain more accurate results.

In order to prepare tablet solution for qNMR, 20 mg ± 0.01 mg commercial rosuvastatin tablet in powder form was weighed and was put into 5 mm NMR tube. Ellagic acid was added on rosuvastatin tablet powder, and obtained mixture powder was dissolved in 1 mL DMSO- d_6_.

### 2.4. HPLC analysis of rosuvastatin

1 mg ( ± 0.001 mg) rosuvastatin was accurately weighed and dissolved in 1 mL ( ± 0.001 mL) deionized water. Obtained solution was filtered with 0.22 µm membrane filter (GVS, Standfor, ME, USA). This solution was used as the stock solution of standard. Sample solution was prepared using the same method. Obtained solution was diluted with the distilled water for different concentration. The sample and standard solutions were injected to HPLC.

## 3. Results and discussion

**Figure 2:**
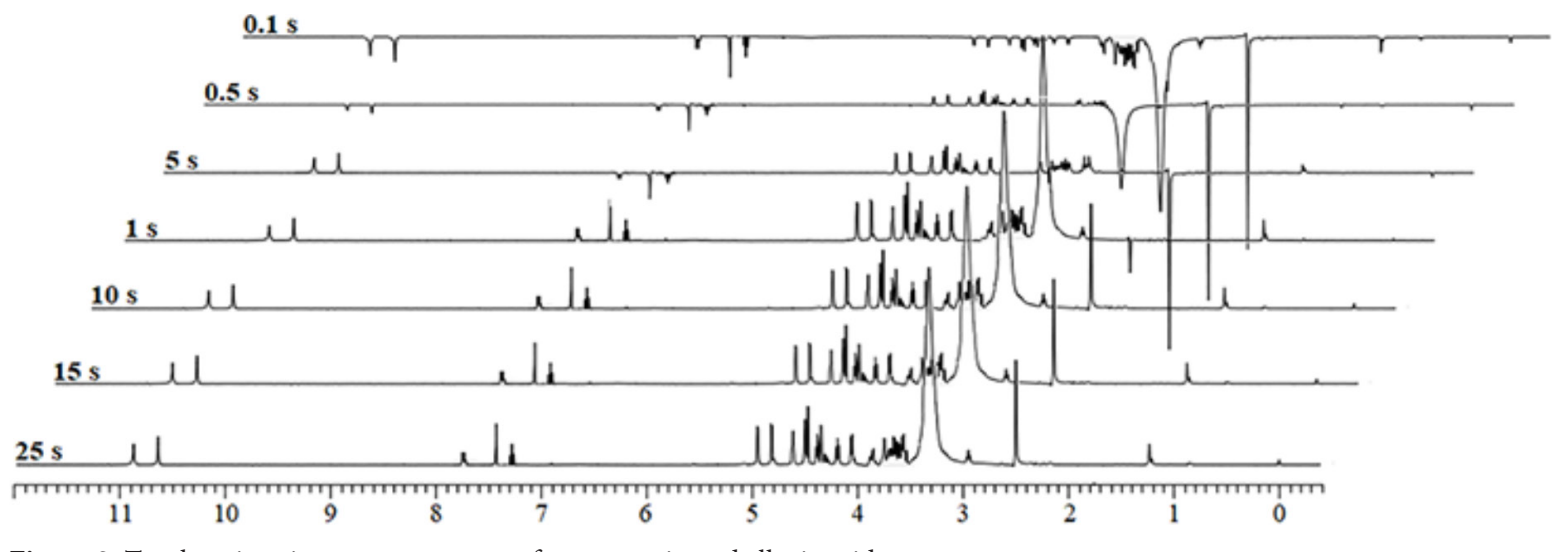
T_1_ relaxation time measurements of rosuvastatin and ellagic acid.

The most important factor in the qNMR method is spin-lattice relaxation time (T_1_). In this study, T_1_relaxation time measurement experiments of rosuvastatin and ellagic acid were performed with inversion recovery method [21]. Obtained 1H NMR signals in relaxation time measurement experiments were given in Figure S1. T_1_relaxation times of signals observed at 6.51 ppm, 4.19 ppm, and 3.54 ppm of rosuvastatin were measured as 1.877 s, 1.991 s, and 1.467 s, respectively. T_1_relaxation time of CH, OH (2), and OH (3) groups of ellagic acid were measured as 2.221 s, 1.596 s, and 1.569 s, respectively, and therefore, the relaxation times were set as 25 s in all^1^H NMR experiments depending on the resulting relaxation times (Figure 2).

### 3.1. Validation

#### 3.1.1. Speciﬁcity and selectivity

**Figure 3:**
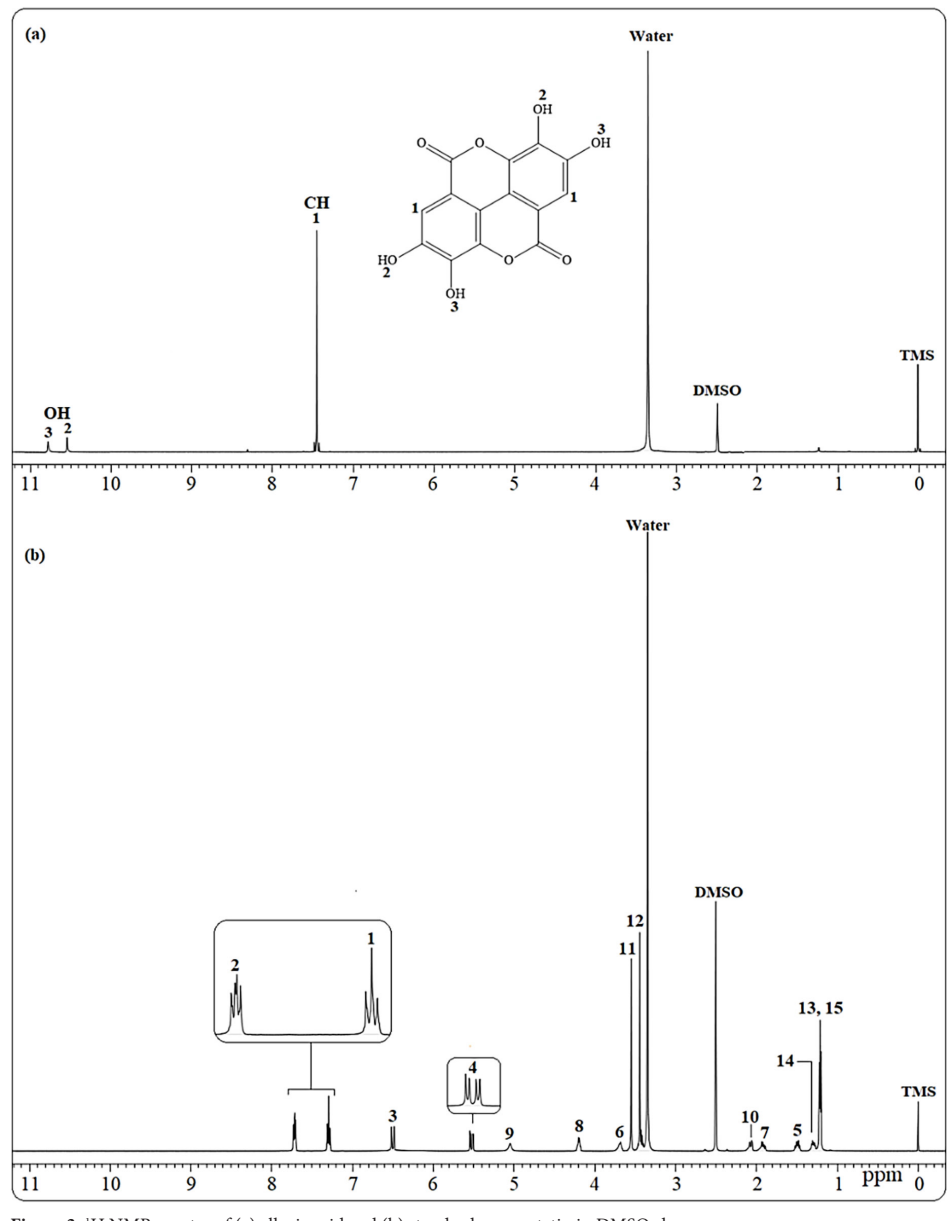
^1^H NMR spectra of (a) ellagic acid and (b) standard rosuvastatin in DMSO-d_6_.

**Figure 4:**
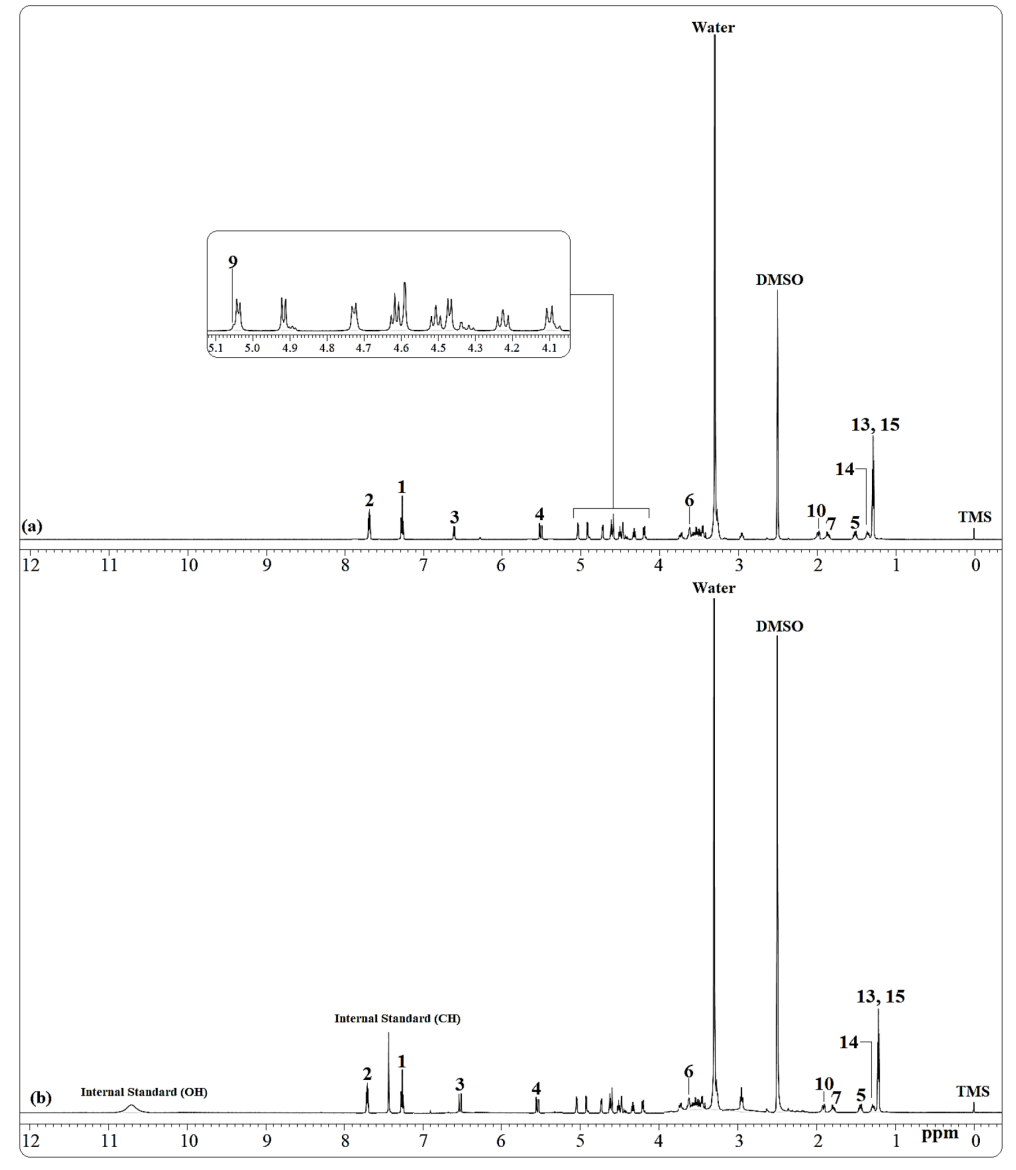
^1^H NMR spectra of (a) rosuvastatin tablet form and (b) rosuvastatin tablet and ellagic acid in DMSO-d_6_

In order to determine interferences in sample solutions, speciﬁcity and selectivity were performed with NMR. Internal standard (ellagic acid), standard sample (rosuvastatin), and tablet of rosuvastatin samples were dissolved in DMSO-d_6_and^1^H NMR spectra were collected using NMR for speciﬁcity study. Obtained spectra are given in Figures 3 and 4. As can be seen from the obtained spectra, NMR signals of internal standard sample, rosuvastatin, and solvent did not overlap with each other. On the other hand, used signals did not affect each other when the qNMR method is used. Obtained spectra shows a convincing speciﬁcity and selectivity of the qNMR method for rosuvastatin validation and optimization studies.

#### 3.1.2. Linearity and range

In order to perform linearity study of rosuvastatin, firstly five different concentration solutions containing rosuvastatin (0.12, 2.14, 2.93, 4.24 and 4.91 mg rosuvastatin were dissolved in 1 mL DMSO-d_6_) were prepared and then 1H NMR spectra of samples with different concentrations were obtained. Calibration graphs in qNMR methods were obtained using a ratio of the analyte to internal standard (IS) for peaks appeared at 6.51 ppm, 4.19 ppm, and 3.54 ppm, and calibration curves were obtained using these ratios with the sample concentration for each signal. The calibration curves are given in Figure S1. The correlation coefﬁcients of quantitative protons observed at 6.51 ppm, 4.19 ppm, and 3.54 ppm were calculated as 0.9947, 0.9944, and 9963, respectively. According to the results obtained, a perfect linearity of the qNMR method was observed. In this study, the calibration graph drawn for 3.54 ppm, which has the best r2 value, was used.

However, since R2 values in other calibration charts are very close to each other, they can be used as a calibration chart. On the other hand, it was observed that there was no difference between the groups because of P > 0.05 in the ANOVA test.

#### 3.1.3. Accuracy

The accuracy study of qNMR method was determined using recovery test. Different amounts of pure rosuvastatin were added into tablet rosuvastatin sample in which quantity of rosuvastatin was known.

The accuracy was determined using the following formula [19]. Moreover, m_s_(weight of rosuvastatin in tablet), m_x_(obtained weight of rosuvastatin), m_0_(weight of added rosuvastatin), and RSD (relative standard deviations) according to 3 different signals were given in Table 1. The average recovery values and RSDs show that the qNMR can provide an ideal accuracy for rosuvastatin determination.

Recovery(%)=mx-m0msx100

**Table 1:** Recovery test results of rosuvastatin.

No	Rosuvastatin					Recovery (%)		
	mo (mg)	ms (mg)	mx (mg)			6.51 ppm	4.19 ppm	3.54 ppm
			6.51 ppm	4.19 ppm	3.54 ppm			
1	0.627	0.805	1.424	1.425	1.421	99.63	99.38	99.50
2	0.629	1.012	1.635	1.640	1.639	99.41	99.90	9980
3	0.641	1.231	1.868	1.862	1.864	99.67	99.51	99.35
4	0.601	1.801	2.415	2.418	2.414	100.72	100.89	100.67
5	0.621	2.310	2.936	2.938	2.933	100.22	100.13	100.87
Average	-	-	-	-	-	99.93	99.96	100.04
RSD %	-	-	-	-	-	1.06	1.02	1.11

#### 3.1.4. Precision

Precision study was determined using RSD of repeatability and inter-day and intra-day precision. The repeatability was tested using three different amounts of rosuvastatin and each experiment was repeated 3 times including sample preparation. Inter-day precision and intra-day precision were shown in Table 2 and Table 3, respectively.

**Table 2:** Inter-day precision of rosuvastatin.

Rosuvastatinamounts(mg)	0th Hour				6th Hour				12th Hour			
	Measure amounts (mg)			RSD %	Measure amounts (mg)			RSD %	Measure amounts (mg)			RSD %
	6.51ppm	4.19ppm	3.54ppm		6.51ppm	4.19ppm	3.54ppm		6.51ppm	4.19ppm	3.54ppm	
0.805	0.804	0.803	0.802	0.12	0.803	0.801	0.799	0.10	0.799	0.798	0.797	0.13
1.231	1.229	1.226	1.227	0.11	1.228	1.230	1.227	0.13	1.227	1.231	1.230	0.16
2.310	2.299	2.304	2.301	0.14	2.302	2.305	2.301	0.16	2.304	2.302	2.305	0.18

**Table 3:** Intra-day precision of rosuvastatin.

Rosuvastatin amounts(mg)	Day 1				Day 2				Day 3			
	Measure amounts (mg)			RSD %	Measure amounts (mg)			RSD %	Measure amounts (mg)			RSD %
	6.51 ppm	4.19 ppm	3.54 ppm		6.51 ppm	4.19 ppm	3.54 ppm		6.51 ppm	4.19 ppm	3.54 ppm	
0.805	0.799	0.797	0.800	0.15	0.796	0.799	0.801	0.10	0.796	0.794	0.797	0.12
1.231	1.226	1.227	1.225	0.13	1.225	1.226	1.224	0.15	1.214	1.213	1.215	0.14
2.310	2.297	2.304	2.305	0.17	2.305	2.303	2.305	0.17	2.284	2.282	2.285	0.19

#### 3.1.5. Limit of detection (LOD) and limit of quantiﬁcation (LOQ)

LOD and LOQ values were determined using standard deviation of the response σ and slope S of calibration curve using following equations.

LOD = 3.3σ / S LOQ = 10σ / S

LOD and LOQ values were calculated as 0.25 mg/mL and 0.80 mg/mL, respectively.

#### 3.1.6. Robustness

Robustness refers to the effect of experimental results caused by the variations acquisition parameters. In this study, robustness was assessed on the difference analysis from the single variable test of the following acquisition parameters: the number of scans, the relaxation delay, the pulse width, the data points, and spectral width. Robustness results are given in Table 4, and robustness results show that analysis parameters do not signiﬁcantly change the results compared with the optimized state.

**Table 4:** Robustness results of rosuvastatin.

Parameters	Optimized	Changed values	Difference (%)		
			6.51 ppm	4.19 ppm	3.54 ppm
Number of scan	16	8	0.21	0.22	0.25
		32	0.24	0.25	0.29
Number of prescan	4	0	0.29	0.28	0.25
		8	0.33	0.35	0.27
Data points	16K	8K	0.42	0.37	0.34
		32K	0.41	0.39	0.39
Spectral width	20 ppm	15 ppm	0.50	0.57	0.52
		25 ppm	0.52	0.55	0.54
Relaxation time	25 s	5 s	0.31	0.36	0.35
		25 s	0.33	0.39	0.31

#### 3.1.7. The determination of rosuvastatin in tablet

Amount of rosuvastatin in the tablet was determined by the qNMR method and by HPLC. Obtained results were given in Table 5. A difference was observed between the results obtained from two different signals used in the qNMR method. A difference was observed between the results obtained from two different signals used in the qNMR method. Depending on this situation, their interaction with other protons in the environment or their interaction with the solvent is also different. Therefore, the T1 relaxation times are also different from each other. As a result, the difference in the relaxation times can cause different amounts of rosuvastatin in tablet form. In addition, the results obtained by the NMR method and the results obtained by HPLC are the perfect match, and the results obtained by both methods are similar.

**Table 5:** The determination of rosuvastatin using qNMR method and HPLC.

Sample	qNMR (n = 3)			HPLC (n = 3)	
	Signals (ppm)	%	RSD%	%	RSD%
Rosuvastatin 5mg tablet	6.51 ppm	100.01	0.54	100.7	0.5
	4.19 ppm	99.56	0.48		
	3.54 ppm	99.92	0.45		

Amount of rosuvastatin in the tablet was calculated using the following formula [9,20]:

%Rosuvastation=IxIstdxNstdNxxMxMstdxmstdmtabletxTLxPstdx100

In this formula, I_x_and I_std_show the integrated value of rosuvastatin and ellagic acid. N_x_and N_std_show the number of protons of used quantitative signals of rosuvastatin and ellagic acid. M_x_and M_std_indicate the molecular weight of rosuvastatin and ellagic acid. mtablet and mstd denote the mass of rosuvastatin and ellagic acid. T and L are average tablet weight and amount of rosuvastatin in the tablet, respectively. P_std_is the purity of ellagic acid.

### 3.2. HPLC analysis of the rosuvastatin in the tablet

**Figure 5:**
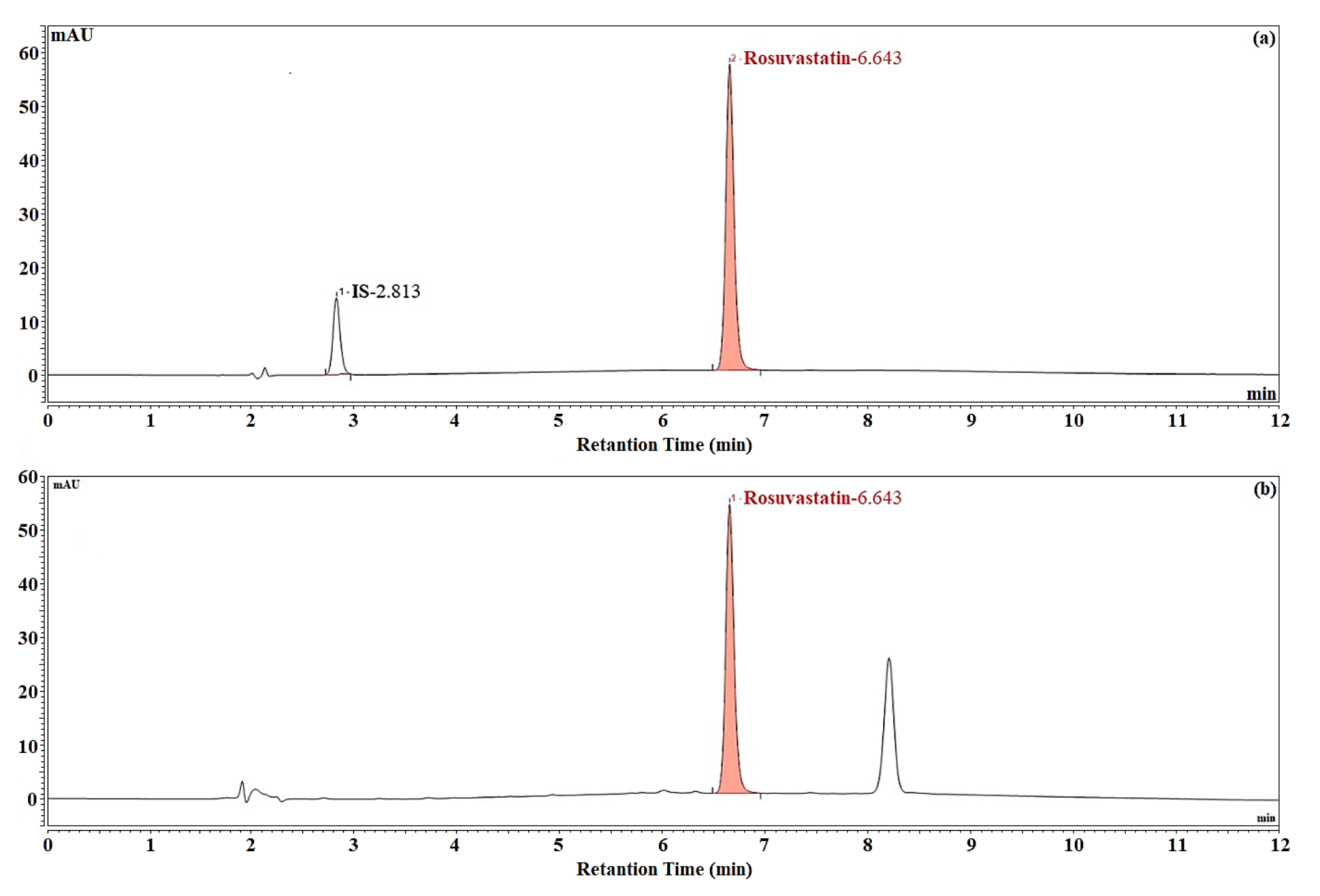
HPLC chromatogram of rosuvastatin (a) and rosuvastatin tablet (b).

The method was validated in accordance with International Conference on Harmonisation of Technical Requirements for Registration of Pharmaceuticals for Human Use (ICH) [22]. One tablet containing rosuvastatin was weighted, and then tablet was powdered in tablet mortar. 1 mg well-mixed powder was weighted from tablet containing 5 mg rosuvastatin and transferred into a 2 mL vial. A total of 2 mL distilled water and caffeic acid as internal standard material were added into vial, and the mixture was sonicated for about 12 min. HPLC chromatograms of rosuvastatin tablet were given in Figure. 5.

Amount of rosuvastatin per unit dose was calculated using fallow formula [17];

mx=AxAstdxmstdmtabletxPstdxT

where m_tablet_: powder is the weighted mass of tablet powder sample, T is weight of tablet, P is purity of rosuvastatin.

Linearity was investigated in the range of 2.0 × 10^-9^- 1.99 × 10^-7^M for rosuvastatin. The coefficient of variation of inter-day experiments for calibration experiments were 0.9992 for rosuvastatin. Calibration graph of rosuvastatin was given in Figure S2.

For the precision of the method, a standard solution containing 1, 25, and 100 ppm rosuvastatin, and IS were analyzed in three consecutive days with five replicates. The RSD% values were below 2% as seen in Table 6, which shows that the method is precise. Accuracy studies were conducted with three different concentrations for rosuvastatin. Each solution was injected six times a day. The accuracy results of the method are given in Table 7.

**Table 6:** Precision of HPLC method.

Linearity Parameters	Intra day (n = 5)			Inter day(n = 11)
	Day 1	Day 2	Day 3	
Mean	20.108	20.761	20.741	21.105
SD	0.170	0.179	0.171	0.016
RSD%	0.845	0.864	0.829	1.377

**Table 7:** Accuracy of the HPLC method.

Amount of rosuvastatin (ppm)	Found (ppm)	Recovery%	RSD%
1	1.019	101.90	0.74
25	25.135	100.54	0.16
100	101.342	101.34	0.32

The sensitivity of the method was calculated by using the slope of calibration curve and the standard deviation of the response. The limit of detection (LOD) values was calculated as 1.02 × 10^-9^M, and limit of quantification (LOQ) values were calculated as 3.39 × 10^-9^M for rosuvastatin. The selectivity of the method was proved with checking the signals at different wavelengths during analysis. No interferences with the peaks of interest were observed.

### 3.3. Uncertainty evaluation of qNMR and HPLC methods

Uncertainty is very important parameter because uncertainty in the measurement affects the quality and reliability of measured result. The purity of IS and rosuvastatin with reference to tyrosine was calculated in qNMR method. Uncertainty of HPLC method was determined using EURACHEM/CITAC Guide CG 4 (3th edition) quantifying uncertainty in analytical measurement [23, 24]. Uncertainty parameters of qNMR and HPLC methods were given in Tables 8 and 9.

**Table 8:** Uncertainty evolution of HPLC method.

	x	U(x)
Weighing of the starting sample (mg)	10.04	0.0178
Stock solution (mg/ml)	1000	0.7786
Repeatability	1	0.0016
Combined standard Measurement uncertainty (%)	2.012	
Expanded standard Measurement uncertainty (%)	4.014	
Relative measurement uncertainty (%)	0.58	

**Table 9:** Uncertainty evolution of qNMR method.

	x	U(x)
Purity of rosuvastatin (%)	98.00	0.08551
Purity of IS (%)	95.00	0.02717
Purity of reference (%)	99.00	0.00330
Mw of rosuvastatin (g/mol)	500.57	0.02450
Mw of IS (g/mol)		0.03237
Mw of reference (g/mol)	181.19	0.00416
Purity of rosuvastatin (%)	98.00	
Combined standard Measurement uncertainty (%)	0.087	
Expanded standard Measurement uncertainty (%)	0.176	
Relative measurement Uncertainty (%)	0.179	

For qNMR, the standard uncertainty*u*(*P*_x_) can be calculated by the following equation [25]:

u(Px)=Px(u(IxIstd)IxIstd)2+(u(Mx)Mx)2+(u(mstd)Mstd)2+(u(mx)mx)2+(u(mstd)mstd)2+u(PstdPstd)2

*u*(*P*_ref_) was obtained from the certificate of tyrosine, and then*u*(*M*_*x*_) and*u*(*M*_std_) were calculated by the following equation:

u(M)=∑j=1n[Nju(j)]2

Uncertainty of purity measurement was evaluated as follows:

u(mi)=uW2(m)+22unon-linear2(m)

The purity of rosuvastatin measured by qNMR can be calculated by the following equation:

Px=IxIstdNstdNxMxMstdmstdmxPstd

Equation of qNMR method for analyte mass is as follows:

Wx=IxIstdNstdNxMxMstdmstd

I = peak ares of number of proton, N = number of proton, M_std_= molecular weight, m_std_= weighed mass, P_std_= purity, W_x_= mass of analyte.

## 4. Conclusions

In order to determine the content of rosuvastatin in the tablet, qNMR method was developed. Moreover, content of rosuvastatin in the tablet was also determined with HPLC. It was concluded that qNMR method could determine identification and quantification of rosuvastatin. The results obtained according to the calibration graphs obtained from different NMR signals are similar to each other. Observation and interpretation of characteristic 1H NMR signals of rosuvastatin ensures authenticity of these samples. Moreover, qNMR method can use to determine impurities and other components without reference materials. On the other hand, retention time was used to identify the rosuvastatin. qNMR method is an accurate, precise, simple, fast, and repeatable method for rosuvastatin determination. As a result, qNMR method and HPLC method confirmed that both methods indicate reasonable agreement in the determination of rosuvastatin in the tablet. Moreover, qNMR method is an easy, practical, and useful method for determination of rosuvastatin in the tablet form. The content determination results of rosuvastatin tablets were identical when comparing the qNMR method to the HPLC method. Moreover, qNMR method could be used as an alternative to or in addition to chromatographic methods for measurement of rosuvastatin in the tablet.

Supplementary Materials
