# Supplementary material for: The application of qNMR for the determination of rosuvastatin in tablet form
Source: Turk J Chem. 2021 Feb 17;45(1):132–42. doi: 10.3906/kim-2007-7 (PMC7925300; doi:10.3906/kim-2007-7)
Supplement: Supplementary file 1 — Supplementary Materials [file turkjchem-45-132-sup001.pdf]

Supporting Information

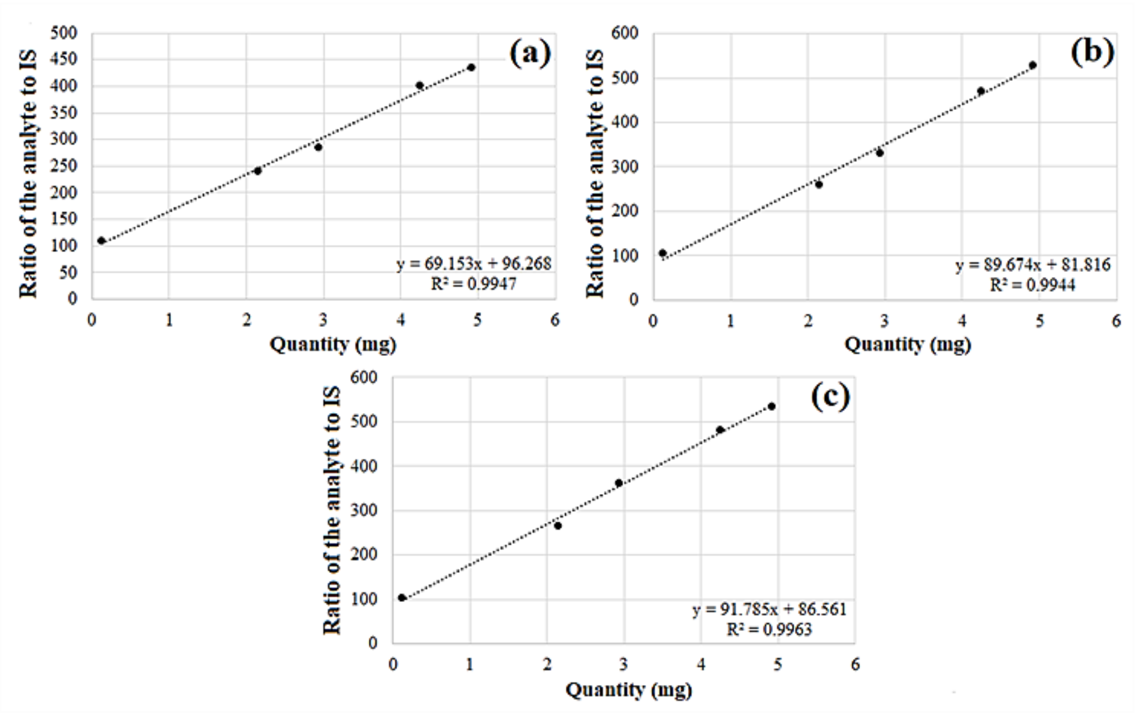

Figure S1. Calibration curves of rosuvastatin (a) for signal at 6.51 ppm, (b) for signal at 4.19, and (c) for signal at 3.54 ppm.

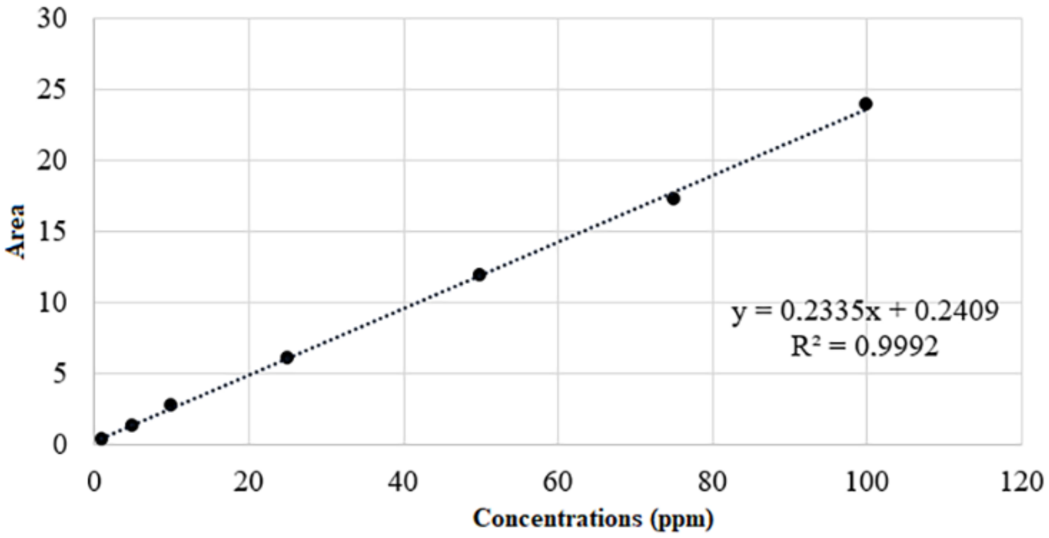

Figure S2. Calibration graph of rosuvastatin.
